# Ultrasound-Guided Dorsal Penile Nerve Block in Children: An Anatomical-Based Observational Study of a New Anesthesia Technique

**DOI:** 10.3390/children11010050

**Published:** 2023-12-29

**Authors:** Markus Zadrazil, Georg Feigl, Philipp Opfermann, Peter Marhofer, Daniela Marhofer, Werner Schmid

**Affiliations:** 1 Department of Anesthesia, Critical Care and Pain Medicine, Medical University of Vienna, 1090 Wien, Austria; markus.zadrazil@meduniwien.ac.at (M.Z.); philipp.opfermann@meduniwien.ac.at (P.O.); peter.marhofer@meduniwien.ac.at (P.M.); daniela.marhofer@meduniwien.ac.at (D.M.); 2 Institute of Anatomy and Clinical Morphology, University Witten/Herdecke, 58455 Witten, Germany; georg.feigl@uni-wh.de; 3 Department of Special Anesthesia and Pain Therapy, Medical University of Vienna, 1090 Wien, Austria

**Keywords:** general anesthesia, pediatrics, anatomy, ultrasonography, dorsal penile nerve

## Abstract

Dorsal penile nerve block stands out as one of the commonly employed regional anesthetic techniques in children. Despite the large body of experience, failure rates are still significant. We included 20 children (median (SD) age of 73 (31) months) scheduled for circumcision without general anesthesia and secondary airway manipulation in a consecutive case series. Under ultrasound guidance and utilizing an in-plane needle guidance technique, the dorsal penile nerve block was administered with slight sedation, and spontaneous respiration was maintained in all cases. To investigate the underlying anatomy for dorsal penile nerve blockade, we dissected three cadavers. The primary study endpoint was the success rate of surgical blockade, meaning that the surgical procedure could be performed without additional general anesthesia and invasive airway management. The secondary endpoint was the requirement of analgesics until discharge from the post-anesthesia care unit. The primary endpoint was successfully met in all patients according to our strict definition without additional general anesthesia or airway manipulation. In addition, no child received analgesics until discharge from the recovery room. The anatomical investigation clarified the specific anatomy as baseline knowledge for an ultrasound-guided dorsal penile nerve blockade and enabled successful performance in 20 consecutive children where penile surgery was possible in light sedation without additional airway manipulation.

## 1. Introduction

Dorsal penile nerve blockade (DPNB) is one of the most frequent regional anesthetic techniques and can be performed for many surgical indications [1,2,3]. Bateman et al. initially described this technique in 1975 [4] and it was refined by Brown et al. in 1989 [2]. Traditionally, DPNB is based on landmark orientation described by Dalens et al. [5]. Ultrasound guidance for dorsal penile nerve blocks have already been described by several groups [6,7]. Very recently, a technically promising ultrasound-guided technique via an in-plane ultrasound needle guidance was described in a short report [8]. Nevertheless, even the ultrasound-guided technique showed a failure rate of 27%, which might be due to the complex anatomy of the fascial layers at the distal abdominal wall. This could be the reason why all DPNB studies so far have been conducted on a population under general anesthesia.

The exact knowledge about topographical anatomy is indispensable for the successful performance of regional anesthesia. “Successful performance” in this context is defined as intraoperative opioid-free management of anesthesia without invasive airway management. We follow the concept of opioid-free anesthesia to the greatest extent since more than a decade ago [9], and the next logical step is focused on effective regional anesthesia for penile surgery.

We therefore designed a “proof-of-concept” observational study where, based on initial anatomical studies, ultrasound-guided in-plane penile nerve blockade was performed in 20 consecutive spontaneously breathing children. A strict definition of block success (no invasive airway manipulation, no intraoperative opioids) was used as the outcome parameter.

## 2. Materials and Methods

### 2.1. Preparations, Enrolling Patients, and Exclusion Criteria

**Figure 1:**
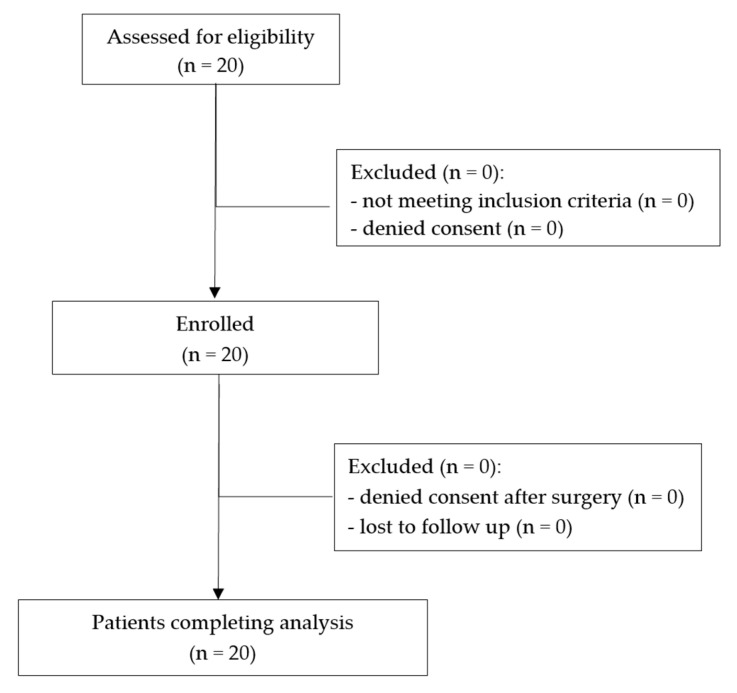
STROBE diagram of study population.

The Ethics Commission at the Medical University of Vienna approved this prospective case series (ref. 1742/2019, approval date 14 July 2020), it was registered into the German Clinical Trial Register (DRKS ID: DRKS00022377, approval date 21 July 2020), and the design of the study adhered to the principles outlined in the STROBE statement (Figure 1). The execution of this study adhered to applicable regulations and guidelines. Written consent was acquired from parents or legal guardians of all participating children, who were thoroughly briefed on the study’s nature, scope, and the procedures to be carried out.

Twenty consecutive children aged 1 to 11 years, who were scheduled for circumcision, underwent eligibility assessment and were subsequently enrolled in our hospital, (Division of Pediatric Surgery, Medical University of Vienna, Austria), from August 2020 to August 2021. Exclusion criteria for the study included the presence of allergies, local infection at the intended site, coagulation disorders, or thrombocytopenia. Additionally, participants who had taken part in another clinical study within the preceding 4 weeks before surgery, those with clinically relevant ECG abnormalities like AV block or bradycardia, or individuals unable to comprehend the study protocol and/or all associated procedures were excluded. Surgical exclusion criteria were not explicitly specified.

### 2.2. Anesthesia Management

The standard care protocol in our department mandates a preoperative fasting period of 6 h for solid food, 4 h for breast milk, and 2 h for clear fluids. If needed, preoperative medication was administered rectally or orally using 1 mg kg^−1^ midazolam (Dormicum^TM^; Roche, Vienna, Austria) with a maximum dose of 15 mg, thirty minutes before the commencement of the procedure. Children below six months of age did not receive premedication. Cardiorespiratory monitoring, including ECG, non-invasive arterial pressure, and SpO_2_, commenced with the child positioned on a forced-air warming blanket (Bair Hugger; Arizant, Eden Prairie, MN, USA). Sedation was started either through a face mask delivering a combination of sevoflurane/oxygen/air or, in the presence of an established vascular access, with a propofol bolus of ≤2 mg kg^−1^, but to minimize airway manipulation and to maintain spontaneous breathing, we preferred sevoflurane sedation, particularly in small children (≤24 months). Based on age, the children were administered an infusion of either 5 mL kg^−1^ h^−1^ Elo-Mel isotone or 10 mL kg^−1^ h^-1^ Elo-Paed (Fresenius Kabi, Graz, Austria) supplemented with glucose 1%. Administration of sevoflurane was halted upon completion of the DPNB. During the surgery, continuous confirmation of spontaneous breathing was ensured using an end-tidal CO_2_ line connected to a face mask secured with adhesive tape, through which oxygen-enriched air (FiO_2_: 0.40) was delivered.

### 2.3. Anatomical Methodology and Investigations

**Figure 2:**
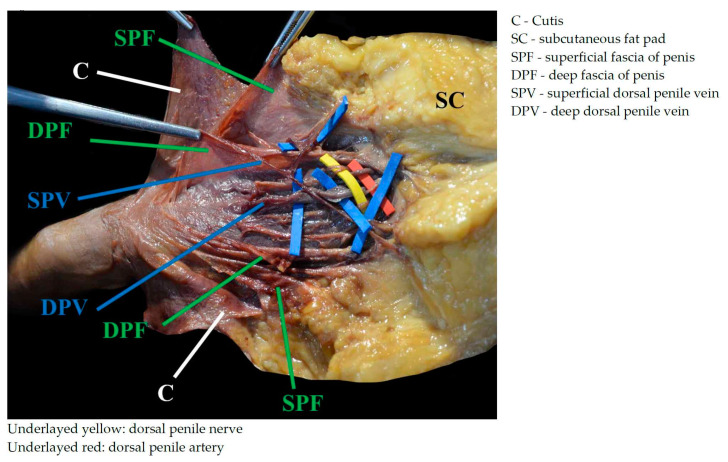
Anatomical presentation.

Apart from the patient case series, three cadaveric specimens fixed with the Thiel’s method [10] were dissected step by step to demonstrate the topographic anatomy of the structures relevant to the ultrasound-guided DPNB. The cadavers were in prone position, and the pudendal region and the dorsum of the penis were meticulously dissected layer by layer. After skin incision by a median incision, the skin was dissected laterally to expose the superficial fascia of the penis, which is a continuation of the deep and superficial layer of the subcutaneous tissue layer [11]. Thiel used the term lamina superficialis and lamina profunda strati subcutanei. On the abdominal wall, these two layers are separated and known as Camper’s and Scarpa´s fascia [12,13]. Scarpa´s fascia corresponds to the deep layer which continues into the pudendal and perineal region as the superficial perineal fascia of Collesi [11,13,14]. On the penis, both layers fuse together, forming the superficial fascia of the penis or superficial penile fascia COLLESI [15]. Underneath the superficial fascia, the space containing the dorsal superficial veins of the penis were identified, dissected, and traced cranially (Figure 2). These veins drain into the external pudendal veins by reaching the subinguinal region. Next, the deep fascia of the penis (deep penile fascia BUCK) was incised to expose the deep dorsal vein of the penis (deep penile vein), the bilaterally running dorsal artery of the penis (dorsal penile artery), and the dorsal nerve of the penis or the dorsal penile nerve (Figure 2), with the nerve being located most laterally of all three structures. The deep dorsal vein of the penis drains inferiorly the symphysis pubica into the vesicoprostatic venous plexus, located in the retropubic space of Retzius. The artery is a terminal branch of the internal pudendal artery and the nerve represents a terminal branch of the pudendal nerve. Deep in the entire deep neurovascular bundle of the penis, the tunica albuginea was identified.

### 2.4. Ultrasound-Guided Dorsal Penile Nerve Block

Following the initiation of sedation, local infiltration of the perineal nerve with 0.5 mL of local anesthetic (LA) was conducted just above the skin of the scrotum. This step was taken because the dorsal penile nerve innervates the glans penis, excluding the area of the frenulum, which is double-innervated by the dorsal penile nerve and a branch of the perineal nerve [16]. Subsequently, the ultrasound probe (Sonosite M-Turbo, Bothell, WA, USA) equipped with a linear array probe (5 to 10 MHz) was positioned in a coronal plane precisely at the location where local infiltration of the perineal nerve was administered above the scrotum. This positioning enhanced ultrasound transmission by optimizing the liquid interface between the penis and the probe. In this perspective, the corpus spongiosum is situated in close proximity to the ultrasound probe. The paired penile neurovascular sheaths are positioned immediately adjacent to the tunica albuginea of the two corpora cavernosa on either side of the midline. 

**Figure 3:**
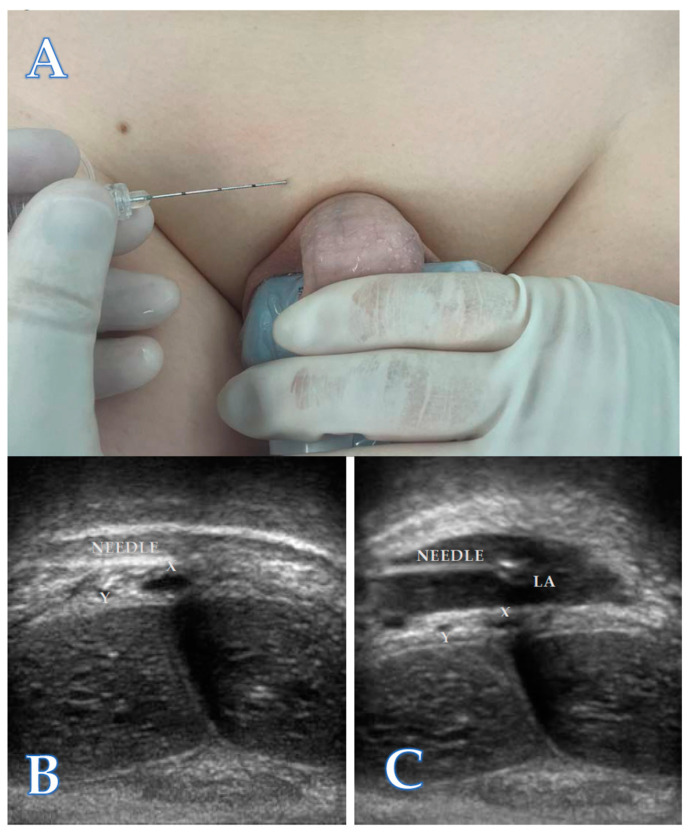
Probe positioning (**A**) and echographic visualization of the ultrasound-guided dorsal penile nerve block before (**B**) and after (**C**) LA injection. Dorsal penile arteries (Y), deep dorsal penile vein (X).

The block was executed utilizing a 22-gauge needle aligned in-plane with the linear array probe positioned in the previously mentioned coronal plane (Figure 3A). Once the penile neurovascular bundle with the dorsal penile nerves and dorsal penile arteries (Y on Figure 3B,C) lateral to the deep dorsal penile vein (X on Figure 3B,C) was visualized, the needle was inserted about 0.5 to 1.0 cm lateral to the mid-sagittal plane, and directed from lateral to medial to approach the penile neurovascular bundle (Figure 3B). Local anesthetic (LA) was injected after negative aspiration. We used bupivacaine 0.5% with volumes of 0.1 mL kg^−1^. The spread of local anesthetic was seen as a black hypoechoic area filling the neurovascular sheath (Figure 3C).

### 2.5. Assessment of Anesthesia and Emergency Preparedness

Following a 10 min onset time for surgical analgesia, the surgeon performed a skin-prick test by applying forceps to the foreskin. If there are movements or a heart rate increase exceeding 15% from the baseline, indicating insufficient blockade, the anesthesiologist may consider enhancing the blockade by administering an intravenous bolus of fentanyl, in a dosage of up to 1 µg kg^−1^. Following this, a further 5 min delay before making the skin incision is recommended. Successful blockade was determined by the absence of a heart rate increase exceeding 15% from baseline and the absence of other pain indicators such as tachypnea during skin incision. Preparedness with advanced airway management equipment was ensured, and respiratory failure, necessitating its application, was characterized by paradoxical ventilation, disappearance of the end-tidal CO_2_ curve, or a decrease in SpO_2_ to below 92%. The stepwise airway management protocol to reinstate sufficient oxygenation included careful face mask ventilation with an inspiratory pressure of less than 10 mmHg and rapid-sequence intubation using propofol 5.0 mg kg^−1^ and rocuronium 1 mg kg^−1^.

### 2.6. Postoperative Management in the Recovery Room

After transfer to the post-anesthesia care unit, the pain status of the children was monitored depending on age and the developmental stage of the child via OPS score in younger children and with the Faces Pain Scale—Revised (FPS-R) in older children. The OPS score evaluates objective behavioral variables, including crying, facial expression, position of the torso and legs, and motor restlessness. Each pain variable is assigned a score on a three-point scale (0 = none, 1 = moderate, 2 = severe), resulting in a maximum cumulative score of 10. The self-report FPS-R is usually possible by 4 years of age; it graphically depicts 6 faces and gives a maximum score of 10. The scores were assessed upon admission to the recovery room and subsequently at 30 min intervals throughout the postoperative hours until the resolution of the DPNB. If the OPS or FPS-R score was ≥4 in two subsequent measurements, depending on age and pain score, the child received intravenous piritramide 0.05 mg·kg^−1^ or nalbuphine 0.1 mg·kg^−1^ or metamizol 10 mg·kg^−1^. A clinical assessment for local infections was conducted, and the administration of systemic analgesics was scrutinized, 24 h after surgery.

### 2.7. Study Endpoints and Data Analysis

The main focus of the study was achieving a successful blockade, defined as not necessitating sequential airway management for spontaneously breathing patients throughout the surgical procedure. Secondary endpoints encompassed the utilization of fentanyl/propofol during the surgery and the administration of postoperative analgesics in both the recovery room and on the ward. The collected data were examined using spreadsheets (Excel 2016; Microsoft, Redmond, WA, USA) and statistical software (Prism 9.4.1 (681); Graph Pad Software Inc., San Diego, CA, USA), with the normal distribution assessed using negative D’Agostino–Pearson testing.

## 3. Results

As illustrated in Figure 1, a total of twenty consecutive children scheduled for circumcision were included in accordance with the inclusion and exclusion criteria (see Materials and Methods) and all 20 patients successfully concluded the study and underwent evaluation. Relevant demographic information for this population is outlined in Table 1. The median duration of the surgery was 20.5 min (IQR: 15.3–27.0).

### 3.1. Block Deficiencies, Anesthesia, and Medication

The primary endpoint of successful blockade was consistently met, with our novel anesthesia management, including sedation and ultrasound-guided DPNB, producing the anticipated outcomes in every patient (Table 1). After positioning, disinfecting and covering the patients, no skin-prick test or skin incision led to any sign of movement or increase in heart rate; no instances of respiratory failure occurred in any patient, obviating the necessity for sequential airway management, as outlined in the Materials and Methods section. Oxygen saturation consistently stayed within a steady range of 97% to 100% throughout both the anesthetic and surgical procedures. In relation to the secondary endpoints, no deficiencies in blockade were observed in any of the children during the skin incision, resulting in the absence of fentanyl and propofol usage intraoperatively and a lack of postoperative analgesic administration in the recovery room or on the ward (Table 1).

### 3.2. Pain Scores and Final Examinations

All OPS and FPS-R scores stayed below five, ensuring that supplementary systemic analgesics were unnecessary throughout the investigation period (Table 1). Assessments for local infection and neurologic function yielded normal results 24 h post-surgery.

## 4. Discussion

Rufini recently described a “reversed” ultrasound-guided DPNB in 40 children undergoing penile urologic surgery in GA with laryngeal masks [8]. Our study presents an innovative approach to anesthesia management for penile surgery in children. This consecutive case series design is valuable in showcasing the viability of new anesthetic techniques tailored to specific surgical procedures [17], serving as a foundation for additional studies and endorsing hypothesis-driven scientific exploration. In this “proof of concept” study of a novel technique involving ultrasound-guided dorsal penile nerve block during sedation for penile surgery, the need for airway manipulation and mechanical ventilation was entirely circumvented. This method caters to spontaneously breathing patients without a secured airway and may serve as a compelling alternative to GA, particularly for patients susceptible to respiratory complications. Moreover, opioid-free anesthesia was successfully attained in 100% of our study population, a significant deviation from several other US-guided DPNB studies where a notable proportion of patients required opioids either intraoperatively or postoperatively (O’Sullivan: 29.4%, Teunkens: 47%, Rufini: 10%) [6,7,8].

In contrast to the landmark-based Dalens’ technique traditionally used by most pediatric surgeons [5], a precise placement of the needle tip is crucial for an efficient block. To make this possible, we preceded the case study with an anatomical examination of the region. Only detailed knowledge of the anatomical circumstances, structures, and communicating spaces allows one to carry out this ultrasound-targeted blockade efficiently and safely. As the dorsal penile nerves are located in a space with loose connective tissue, the needle tip has to be placed in close proximity. While dissecting the neurovascular structures in this space, we could not identify any tissue layers, septa, or other connective tissue structures limiting the spread. This is important for a unilateral needle tip position, either right or left. An injection of a volume above will usually spread to the contralateral side. However, the detection of a unilateral spread is possible only through ultrasound guidance; if so, advancing the needle tip across the midline is adequate, and there is no need for bilateral injection. Another important issue is the space itself, which provides connections to the lesser pelvis, as mentioned above. An additional connection is dorsally underneath the urogenital diaphragm and between the deep and superficial perineal fascia. From an anatomical point of view, even with a spread in these regions, no complications should be expected, which is in line to our clinical observation. With reference to the complex anatomical considerations just mentioned, it is of fundamental importance to be able to visualize all relevant anatomical structures, including the entire needle pathway, on one ultrasound image. The positioning of the ultrasound probe right above the scrotum in a coronal plane, described as the “reverse” approach by Rufini [8], gives the unique opportunity to utilize an in-plane approach and visualize all necessary anatomical structures in one view.

However, the Dalens’ technique, which relies on landmarks, requires substantial amounts of local anesthetic. Complications, such as intravascular injection leading to local anesthetic systemic toxicity (LAST), glans ischemia, hematoma, edema, and nerve or urethral lesions, have been reported with this method [18]. The exclusive method providing secure and high-quality blocks through optimal needle positioning is the ultrasound visualization of anatomical structures. Ultrasound guidance, beyond merely lowering complication rates [19], offers advantages over traditional techniques. These include higher success rates for blocks [19], quicker onset times for blocks [19], reduced total doses of local anesthetics [19], and the ability to assess the spread of local anesthetics [19]. However, DPNBs have significant failure rates [20] and even the promising above-mentioned “reversed” in-plane ultrasound-guided needle DPNB indicated a 27% failure rate in this population of 40 children [8]. The contrast to the failure rate of 0% in our population is remarkable, since it can be assumed that the depth of anesthesia was significantly more pronounced in the group with laryngeal masks, even if no information was given regarding the depth of anesthesia in the Rufini paper. One explanation is that, in our population, the double-innervated frenulum was locally infiltrated with bupivacaine 0.5%, instead of the application of topical Lidocain 2% on the foreskin in the Rufini population. Moreover, the performed anatomical studies on the three dissected cadaveric specimens gave us the confidence and anatomical knowledge to position the needle tip in close proximity to the deep neurovascular bundle of the penis. We also diligently ensured compliance with the onset time, allowing for a minimum of 10 min after conducting the dorsal penile nerve block (DPNB).

Pediatric penile surgery with a DPNB is commonly performed under GA. To mitigate the potential risks of respiratory events, postoperative apnea, and potential neurocognitive issues associated with these substances used in GA, endeavors are undertaken to eschew GA. Instead, “awake” techniques without airway manipulations are employed in the management of subumbilical procedures. Respiratory events constitute the most prevalent critical incidents related to pediatric GA. The utilization of airway management techniques, such as endotracheal tubes or supraglottic airways, during GA is notably linked to an elevated risk of severe respiratory critical events (relative risk up to 3.36, 95% CI: 2.41–4.67) in comparison to patients under sedation with a face mask as the airway interface [21]. Anatomical obstacles, such as a comparatively narrow airway, are significant factors associated with these incidents [22,23,24]. This series does not provide a conclusive answer regarding whether regional anesthesia under sedation presents a more favorable safety profile for these types of procedures compared to general anesthesia, but in this observational study, no aspiration events were noted. However, we acknowledge that the absence of a complication in our observations does not negate the existence of the associated risk. Nevertheless, existing evidence [25] in children suggests that regional anesthetic blocks can be conducted with comparable safety in sedated and awake patients as they can under general anesthesia.

It is important to note that the concept employed in this study does not involve an “awake” technique. Instead, it follows an approach incorporating premedication with midazolam (if needed) and inhalation induction with sevoflurane to establish intravenous access and perform DPNB. Regarding premedication, we already successfully minimized the requirement for midazolam premedication to alleviate preoperative anxiety by employing non-pharmacological, psychological interventions [26]. Regarding the intraoperative course, in our clinical practice, propofol sedation is administered after finishing the block if the operation will last longer than 30 min, which was not the case in our study, with a median surgery duration of 20.5 min; therefore, no propofol was administered or needed (Table 1). We recognize the possibility of a smooth transition between sedation and general anesthesia, with the primary reliance on the judgment of the attending anesthetist. In this context, the adequacy of sedation was determined when the patient was unconscious and could be aroused only with significant physical stimulation. Despite this, spontaneous respiratory drive was maintained in all cases, and no airway instrumentation was necessary.

Despite being the subject of considerable controversy, several observational studies, albeit with small effect sizes, have explored the correlation between exposure to GA and neurodevelopmental outcomes in humans [27,28,29,30]. Several of these studies have confirmed links between such exposure and negative outcomes, indicating the possible neurotoxic effects of different general anesthetics in infants and young children. Very recently, the impact of GA on cerebral white matter in the developing brain was emphasized [31]. The connectivity established by white matter tracts between brain regions is essential for integrating and coordinating neural activity, playing an important role in cognitive and behavioral functions. Thereby, white matter abnormalities have been linked to diminished mobility and reduced cognitive performance. Infants who underwent anesthesia and surgery exhibited a decrease in whole-brain white matter volume, when compared to control subjects [32]. In children subjected to recurrent general anesthesia procedures, increased exposure to propofol and extended cumulative durations of anesthesia were linked to compromised white matter integrity, particularly in the corpus callosum [33]. In straightforward terms, in our everyday clinical practice, our primary mission must be to alleviate fear, instill confidence, and administer anesthesia safely. However, as long as there is even the slightest suspicion that the medications we use may pose a risk, our foremost objective must be to minimize the quantity of these medications administered or, if possible, avoid them entirely! Furthermore, in certain pediatric age groups, many sedative and analgesic drugs are still utilized off-label [34].

In summary, to be succinct and encapsulate our findings, we firmly believe that, particularly in pediatric anesthesia, simplicity is key: fewer (partially off-label) medications and less invasive airway manipulation. Over the past 15 years, our team has been influenced by these considerations, leading us to embrace the intriguing concept of employing ultrasound-guided locoregional blocks under sedation while maintaining spontaneous breathing in infants and children [9]. Ultrasound guidance for dorsal penile nerve blocks was described by several groups [6,7,8], but with the exception of the work by Rufini, all descriptions of ultrasound-guided blockade differ from the method described in our study, which we detail not just sono-anatomically but also anatomically, based on cadaver studies on three cadaveric specimens. Additionally, all those publications and descriptions, including that of Rufini, are performed in a population under general anesthesia and in all of these studies, a considerable proportion of DPBN patients required opioids, unlike our study, where no patient required any opioids either intraoperatively or postoperatively.

## 5. Conclusions

Performing pediatric penile surgery under ultrasound-guided dorsal penile nerve block in sedated and spontaneously breathing children has the potential to minimize airway manipulation and may also allow for reduced use of GA drugs. The limitations of this study, which could impact the outcomes and our conclusions, include the small sample size, potential uncontrolled factors, and the subjective assessment of sedation levels. Despite the inherent limitations of this investigation and the study design, a proof of concept has been demonstrated, laying the foundation for further investigations. This study serves as an initial step, highlighting the feasibility of pediatric penile surgical interventions in spontaneously breathing patients, provided that the penile root block is executed precisely with a thorough understanding of all anatomical structures and potential challenges; further studies are needed with a higher number of patients to answer the question of whether this approach can be performed adequately safely.

## Figures and Tables

**Table 1:** Demographic and treatment-related data.

Age (Months)	73	(31)
Weight (kg)	24	(19–31)
Height (cm)	116	(105.8–129.5)
Duration of surgery (min)	20.5	(15.3–27.0)
Total volume of bupivacaine 0.5% (mL)	3.0	(2.5–4.0)
Opoid-free (n)	20	
Metamizol (mg)	0	
Nalbuphine (mg)	0	
Piritramid (mg)	0	
Fentanyl (µg)	0	
Propofol (mg)	0	

Data are mean (SD) or median values (interquartile range).

## Data Availability

The data presented in this study are available on request from the corresponding author. The data are not publicly available due to privacy concerns.
